# Shape and Size Variations of Distal Phalanges in Cattle

**DOI:** 10.3390/ani14020194

**Published:** 2024-01-07

**Authors:** Nicoleta Manuta, Buket Çakar, Ozan Gündemir, Mihaela-Claudia Spataru

**Affiliations:** 1 Institute of Graduate Studies, Istanbul University-Cerrahpasa, Istanbul 34320, Türkiye; nicoleta.manuta@mail.ru (N.M.); buketcakar4@gmail.com (B.Ç.); 2 Department of Anatomy, Faculty of Veterinary Medicine, Istanbul University-Cerrahpasa, Istanbul 34320, Türkiye; 3 Department of Public Health, Faculty of Veterinary Medicine, Iasi University of Life Sciences, 700490 Iasi, Romania; mspatarufmv@yahoo.com

**Keywords:** allometry, bovine, centroid size, digital bones, geometric morphometry, Procrustes distance

## Abstract

**Simple Summary:**

There are a total of eight distal phalanges in cattle, including those in the hindlimb and forelimb. These bones exhibit a similar shape. In this study, bones from the same breed of cattle were used to examine whether there were statistical differences in their shapes using the geometric morphometry method. For this purpose, bone samples were analyzed in 3D, with 176 semi-landmarks used for each bone in the study. Landmark operations were performed through automated landmarking via point cloud alignment and correspondence analysis. In the shape analysis, efforts were made to determine shape differences specific to forelimb–hindlimb and medial–lateral bones. The distal phalanges showed subtle shape variations, and it was found that centroid size provided a clearer separation between the bones. The study results provide valuable information that can be referenced in terms of veterinary anatomy.

**Abstract:**

Studies on the structure of the distal phalanx help explain the development of laminitis. Additionally, examining the structure of the distal phalanx from a taxonomic perspective also contributes to veterinary anatomy. In this study, we examined shape variation in the medial and lateral distal phalanx of both fore- and hindlimbs using the geometric morphometry method. We investigated whether the shape of the distal phalanx differed between phalanx positions and how much of the shape variation in this bone depends on size. For this purpose, distal phalanges from 20 Holstein cattle were used, and the bones were digitized in 3D. A draft containing 176 semi-landmarks was prepared for shape analysis, and this draft was applied to all samples using automated landmarking through point cloud alignment and correspondence analysis. A principal component analysis was performed to obtain general patterns of morphological variation. The centroid size (CS) was employed as an approximation of size. Although distal phalanx groups generally showed close variations, PC1 statistically separated the hindlimb lateral distal phalanx (HL) and the forelimb medial distal phalanx (FM) from each other in shape. While PC2 separated HL from other distal phalanx groups, PC3 separated fore- and hindlimb groups. The shape (Procrustes distance) of the hindlimb medial distal phalanx (HM) is markedly less variable than the other three phalanges. The smallest distal phalanx in size was HL. For both forelimb and hindlimb, the medial distal phalanges were larger than the lateral ones. Size (CS) was found to have an effect on PC1 and PC3. In this study, a reference model of the same breeds for distal phalanx was created. These results can provide useful information, especially in terms of veterinary anatomy, zooarchaeology, and paleontology.

## 1. Introduction

Ruminants, constituting the most taxonomically diverse family of large terrestrial mammals currently found, encompass over 140 species spread across a large part of the world [1]. These animals inhabit diverse environments, ranging from tropical forests to desert landscapes [2,3,4,5]. The diversity in bone shapes among ruminants living in these different habitats and climates has not yet been fully determined. In recent years, studies examining the morphometric structures of the distal phalanx have been undertaken [6,7,8]. These studies contribute to the literature’s knowledge in terms of taxonomy and help explain the variations in the distal phalanx.

The number of digit skeletons (ossa digiti manus) varies among animal species, and the number of digits differs between ruminants; in cattle, it is two. Each digit consists of the distal phalanx, medial phalanx, and proximal phalanx [9]. The distal phalanx is the lowest digit bone [10]. In its proximal part, there is the processus extensorius, to which the extensor muscle attaches. Within the hoof capsule, the distal phalanx is suspended from the wall by laminar attachments and supported above the sole by the digital cushion [11].

Various biological processes create morphological diversity among individuals or their parts due to factors such as disease, injury, mutation, and genetic development [12]. Shape analysis is an approach to understanding this morphological diversity [13]. Geometric morphometric methods, based on statistical tools, are used for shape analysis. In this method, anatomical landmark positions on the biological shape are collected, and the anatomical points are evaluated with principal component analysis, highlighting the vectorial changes that are most effective in shape analysis [12]. Apart from size, location, and orientation, landmarks can be analyzed using various statistical techniques; thus, it was observed that variables are based solely on morphology [14]. Geometric morphometric analysis abstracts biological shape through the positioning of two- or three-dimensional landmarks. The resulting shape data are most commonly reduced in dimensionality through principal component analysis. In recent years, 3D shape analysis has become much more readily available [15,16,17], and landmarking automation has enabled the reliable collection of standardized sets of landmarks [18].

Foot diseases in cattle are a common veterinary concern, causing lameness and affecting milk yield or reducing reproductive performance, resulting in significant economic losses [19]. Anatomical differences between the medial and lateral distal phalanges can exacerbate and complicate these issues [20]. In this case, knowing the anatomy of the region better makes a significant contribution to morphological evaluation. Additionally, distinguishing the distal phalanx group is important not only for anatomy but also for the fields of zooarchaeology and paleontology. To better understand shape variation in the context of diseases, we have previously performed various studies investigating the distal phalanx using morphometric comparisons [11,21]. The results obtained contribute to reference information and help explain the anatomical variations of the region. Unlike previous studies, in this study, we examine shape variation in the medial and lateral distal phalanx of both fore- and hindlimbs using the geometric morphometry method. We investigated whether the shape of the phalanx distalis differed between phalanx positions and examined how much of the shape variation in this bone depends on size.

## 2. Materials and Methods

### 2.1. Samples

In this study, the distal phalanges of 20 one-year-old Holstein cattle were used. To ensure the homogeneity of the research, only male cattle (bulls) were used in this study. Samples were collected from slaughterhouses after the animals were slaughtered. Once the distal phalanges were obtained as bones, they were classified as follows: forelimb left lateral distal phalanx (FLL), forelimb left medial distal phalanx (FLM), forelimb right lateral distal phalanx (FRL), forelimb right medial distal phalanx (FRM), hindlimb left lateral distal phalanx (HLL), hindlimb left medial distal phalanx (HLM), hindlimb right lateral distal phalanx (HRL), and hindlimb right medial distal phalanx (HRM).

### 2.2. Data Collection

**Figure 1:**
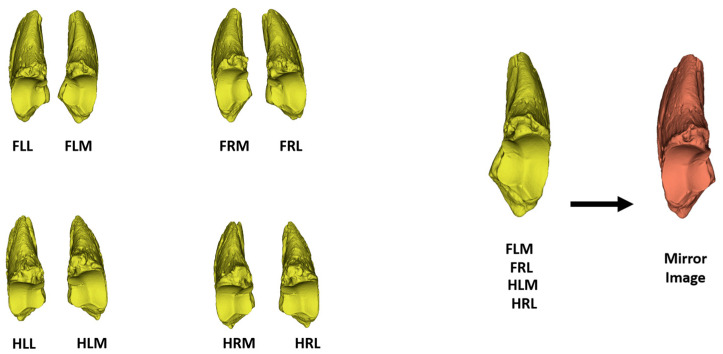
Phalanx distalis used in the study; mirror image for FLM, FRL, HLM, and HRL. FLL—forelimb left lateral distal phalanx; FLM—forelimb left medial distal phalanx; FRL—forelimb right lateral distal phalanx; FRM—forelimb right medial distal phalanx; HLL—hindlimb left lateral distal phalanx; HLM—hindlimb left medial distal phalanx; HRL—hindlimb right lateral distal phalanx; and HRM—hindlimb right medial distal phalanx.

Distal phalanges were digitized using a Shining EinScan-SP scanner with a camera resolution of 1.3 megapixels (DC 12-volt, 3.33 ampere). A resolution of 1280 × 720 was applied to scan these hoofs. EXScan (version 3.1.2.0) was utilized for modeling and merging the scanned images. After the modeling process, the samples were saved as PLY files. Since the automatic landmark system would be used in the study, the posture of all bones had to be the same. Therefore, mirror images of FLM, FRL, HLM, and HRL were taken (Figure 1). Following this process, the samples were examined in four groups: forelimb lateral distal phalanx (FL), forelimb medial distal phalanx (FM), hindlimb lateral distal phalanx (HL), and hindlimb medial distal phalanx (HM). The Slicer program (version 5.5.0) was used for these operations [22].

**Figure 2:**
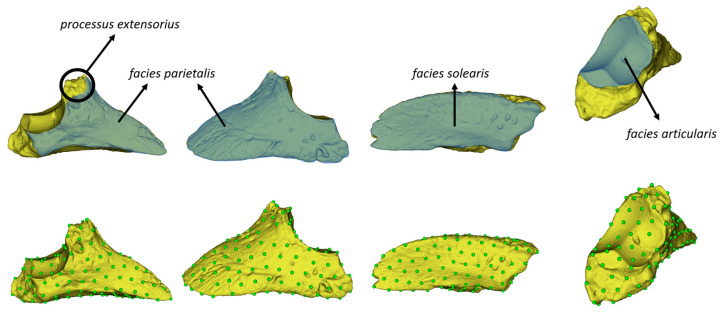
Anatomical terms and landmarks. Draft landmark (176 semi-landmark) model obtained using the PseudoLMGenerator module. Figure taken from ALPACA (automated landmarking through point cloud alignment and correspondence analysis).

For automatic landmark operations, a draft landmark was created initially. The module ‘PseudoLMGenerator’ in ‘3D Slicer’ (V 5.5.0) was used to generate an atlas of 176 equidistant surface semi-landmarks, applying a spacing tolerance of 6% (Figure 2) [23]. Automated landmarking through point cloud alignment and correspondence analysis (AL-PACA) was used to apply these semi-landmarks to other models. ALPACA facilitated rapid landmark transfer from a 3D model and its associated landmark set to target 3D model(s) through point cloud alignment and deformable mesh registration [24]. ALPACA is also a module of the ‘3D Slicer’ (V 5.5.0) program. Batch processing was used to apply the draft pseudo-landmark to all samples. As a result of these operations, files with 176 landmark coordinates were obtained for each sample. The landmarks used are shown in Figure 2.

### 2.3. Size, Shape Variables, and Allometry

Principal component analysis (PCA) was performed to derive general patterns of morphological variation [25]. The PCA was analyzed through the covariance matrix of the Procrustes coordinates obtained after the Generalized Procrustes Analysis (GPA) of the original reference coordinates. For this process, GPA in the SlicerMorph module of the Slicer program (V 5.5.0) was used. As a result of PCA, shape variations of the phalanx distalis were revealed. The distribution among groups was evaluated according to PC1, PC2, and PC3. Centroid size (CS) was employed as an approximation of size. Additionally, Procrustes distance values of the samples were also obtained. CS and Procrustes distances were obtained for all eight phalanges through Slicer. Both of these metrics were used to assess differences between phalanges using a parametric t-test in ‘PAST’ (V 4.14) [26]. The regression of the Procrustes distances against CS on pooled within-group variation (pooled by four groups: forelimb lateral distal phalanx, forelimb medial distal phalanx, hindlimb lateral distal phalanx, and hindlimb medial distal phalanx) was performed to evaluate the allometric effect (the relationship between size and shape) in ‘PAST’ (V 4.14).

## 3. Results

### 3.1. Size and Shape

**Figure 3:**
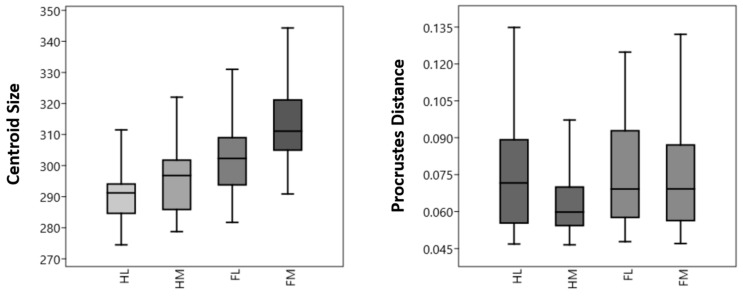
Boxplot with variation in Procrustes distance and centroid size values for distal phalanx groups. The darker horizontal line is the median, the margins of the boxes represent the percentiles (25 and 75), and the extensions of the bars represent the maximal and minimal values for distal phalanx groups. FL—forelimb lateral distal phalanx; FM—forelimb medial distal phalanx; HL—hindlimb lateral distal phalanx; HM—hindlimb medial distal phalanx.

CS values for the distal phalanx are presented in Figure 3. According to the results, it was shown that the forelimb (mean: 281.71 ± 13.15) had higher values than the hindlimb (mean: 274.50 ± 9.33). This difference was statistically significant (F: 1.99, p: *p* < 0.001). For both fore- and hindlimbs, the medial distal phalanges were larger than the lateral ones. In the forelimb, the average CS value of the lateral distal phalanges was 301.87 ± 11.58, while the average CS value of the medial distal phalanges was 312.75 ± 12.49. This difference was statistically significant (*p* < 0.001). In the hindlimb, the average CS value of the medial distal phalanges was 295.64 ± 9.86, while the average CS value of the lateral distal phalanges was 290.09 ± 7.89. The difference between these two bones was statistically significant for the forelimb (*p* < 0.01), with the difference being greater in the forelimb than in the hindlimb. For both fore- and hindlimb, the medial distal phalanges were larger than the lateral ones.

In Procrustes distance results, the HL and FM showed more variation than other bones. Their standard deviations were higher than those of other samples. The shape of the HM is markedly less variable than the other three phalanges. The shape (Procrustes distance) of the HM was statistically different from the HL (P: 0.024), FL (P: 0.034), and FM (P: 0.048). Except for the HM, the shape differences between the other groups were statistically insignificant.

### 3.2. Shape Variation

**Figure 4:**
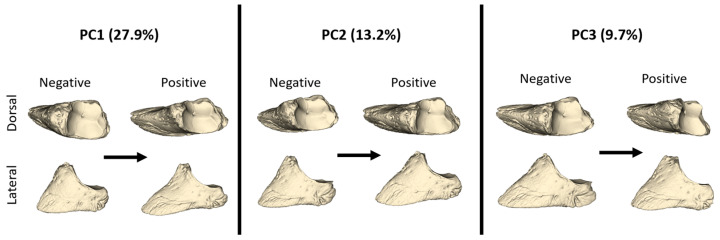
Models describing distal phalanx shape between the negative and positive values of PC1, PC2, and PC3 from lateral and dorsal views.

Negative and positive shape variations according to the PC1, PC2, and PC3 values are given in Figure 4. An increasing PC1 value represented a distal phalanx that was thinner and longer in shape. The facies articularis was narrower at a positive PC1 value. At a negative PC1 value, the facies parietalis had a lesser inclination angle against the dorso-ventral axis, while as the PC1 value increased, the facies parietalis had a higher inclination angle. With these features, a positive PC1 value represented a distal phalanx with a more pointed front. The increased PC2 value also represented a thinner and longer distal phalanx. At a negative PC2, the facies parietalis was quite steep. With an increasing PC3 value, the facies articularis was quite narrow in shape. Additionally, with an increasing PC3 value, the processus extensorius was more developed.

**Figure 5:**
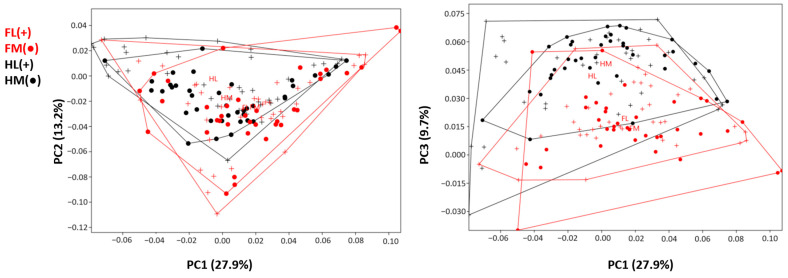
Principal component analysis scatter plot comparing distal phalanx morphology of four groups: forelimb lateral distal phalanx (FL); forelimb medial distal phalanx (FM); hindlimb lateral distal phalanx (HL); and hindlimb medial distal phalanx (HM).

PC1 and PC2 explained 41.1% of the total variation (Figure 5). The mean PC1 value of the forelimbs was higher than that of the hindlimbs, but the shape variations were close to each other. For PC1, it was observed that the groups were not separated from each other. However, hindlimbs can be said to have a lower PC1 value (Table 1). In particular, some samples of HLs had the lowest PC1 values, while FM samples had the highest PC1 values. FM samples also exhibited low PC2 values.

PC3 explained 9.7% of the total variation. Only one HL sample showed a negative PC3 value, while all remaining hindlimb samples had positive PC3 values. The distinction between fore- and hindlimb was more evident in PC3 than in other PCs. Hindlimb samples had high PC3 values. According to these results, the hindlimb distal phalanges were shorter and blunter in shape.

PC3 explained 9.7% of the total variation. Only one HL sample showed a negative PC3 value, while all remaining hindlimb samples had positive PC3 values. The distinction between forelimb and hindlimb was more evident in PC3 than in other PCs. Hindlimb samples had high PC3 values. According to these results, the hindlimb distal phalanges were shorter and blunter in shape.

The mean values of PC1, PC2, and PC3 are presented in the table. For PC1, only the value between the HL and FM was statistically significant (*p* < 0.05). The HL had a negative PC1 value, while the other groups had positive PC1 values. On average, forelimb (FL and FM) PC1 values were higher than hindlimb values. Regarding PC2, the mean values of all groups were negative. The HL had the highest PC2 values. The differentiation of the HL from other bones in terms of shape variation, according to the PC2 value, was statistically significant (*p* < 0.05). PC3 shape variation statistically separated the forelimb distal phalanges from the hindlimbs (*p* < 0.01). Hindlimb samples had high PC3 values, and the group with the highest PC3 mean value was the HM.

### 3.3. Allometry

**Figure 6:**
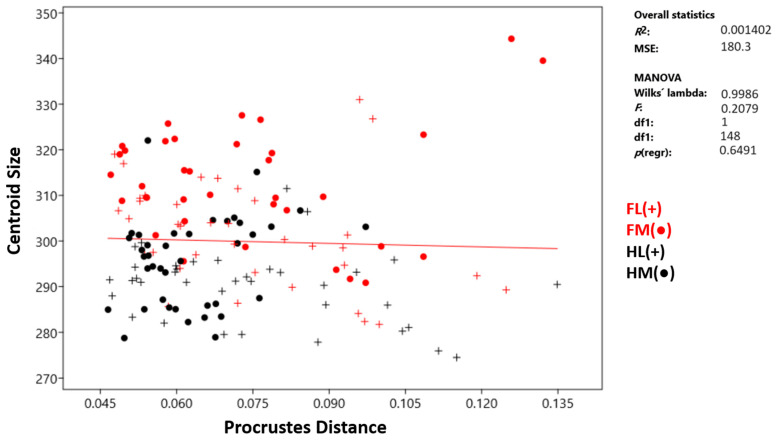
Allometry for distal phalanx. FL—forelimb lateral distal phalanx; FM—forelimb medial distal phalanx; HL—hindlimb lateral distal phalanx; HM—hindlimb medial distal phalanx.

**Figure 7:**
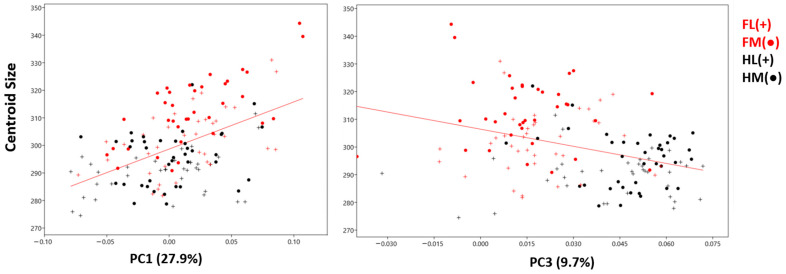
The effect of size (CS) on PC1 and PC3. FL—forelimb lateral distal phalanx; FM—forelimb medial distal phalanx; HL—hindlimb lateral distal phalanx; HM—hindlimb medial distal phalanx.

Multivariate regression was conducted to examine the effect of size (CS) on shape (Procrustes distance). However, allometry was found to be statistically insignificant (Figure 6). Nonetheless, size (CS) was found to have an effect on PC1 (r^2^: 0.499, *p* < 0.001). There was a positive regression between PC1 and size (Figure 7). According to these results, the facies parietalis was more upright as its size increased. Additionally, with increasing size, the distal phalanges had a thinner and longer shape. The effect of PC2 (r^2^: 0.005, *p*: 0.382) on size was statistically insignificant. The effect of size on PC3 was significant (r^2^: 0.126, *p* < 0.001). However, the relationship between PC3 and size was negative. In the shape variation, the positive change in PC1 was similar to the negative change in PC3.

Size (CS) was found to have an effect on PC1 and PC3 (Table 1). According to these results, as the size increases, the distal phalanx becomes thinner and longer in shape. Hindlimb centroid size values with lower PC1 values were generally lower than forelimbs with higher PC1 values. Conversely, hindlimb samples with high PC3 values also had low centroid sizes.

## 4. Discussion

In this study, the geometric morphometric method was employed to distinguish bovine phalanx distalis from each other. Predictably, size (CS) values were found to vary among phalanges groups, with forelimb bones being larger than hindlimb bones (Figure 3). Furthermore, both hindlimb and forelimb medial distal phalanges were larger than the lateral ones. Notably, the HM had a more conservative Procrustes distance than other samples. Although size had no significant effect on Procrustes distance in terms of allometry, it was observed to influence PC1 and PC3 values.

Both Muggli [20] and Ocal [27] found that the distal phalanges of the forelimb are dorsoventrally taller than those of the hindlimb. Additionally, in Gundemir’s [28] study, where he measured the distal phalanx lengths linearly, he reported that the forelimb distal phalanges are longer than the hindlimbs, with the inner side being longer than the lateral ones. In this study, size was evaluated using centroid size values through the geometric morphometry method. The centroid size values were consistent with previous studies on cattle digital bones.

In dairy cows, the forelimb carries more weight than the hindlimbs [29]. In Van der Tol’s [29] study on dairy cows, he investigated vertical ground reaction force and pressure distribution. The maximum vertical ground reaction force, averaged over the left and right limbs, was less in the hindlimb (2444 Newton) than in the forelimb (3324 Newton). These forces accounted for 37% and 51% of the body weight, respectively. The distal phalanges bear the entire weight of the body in both the fore- and hindlimb. It is conceivable that this weight difference between the fore- and hindlimb may contribute to morphological differences in bone shape. This study on the distal phalanges revealed that the bones of the forelimb were larger. When considering general shape variations, the bones of the forelimb exhibited a larger surface area (Figure 3). It can be thought that this morphological difference is related to the forelimb carrying more weight. To explain this in more detail, future studies could examine the effect of the animal’s body weight on shape and centroid size.

During the walking of ruminants, both the lateral and medial distal phalanges share the total weight on the foot [30]. However, when ruminants stand, the medial distal phalanges carry most of the load [30]. Consequently, the medial distal phalanges are generally exposed to more weight, potentially causing asymmetric variation between the two bones during development [31]. In this study, although the medial and lateral distal phalanges exhibited similar shape variations, the lateral ones presented lower PC1 values (Figure 5). It was observed that the structure of these bones with low PC1 samples was narrower in shape, and the surface areas in contact with the ground were shorter. This difference may be attributed to the excess weight falling on the medial distal phalanges. Additionally, when examined in terms of size, it was noted that the medial distal phalanges were larger than the lateral ones. These results support the idea that the distal phalanges may exhibit shape variations due to their greater exposure to weight. However, one of the most important limitations of this study on the distal phalanx was the lack of information about the weight of the animals. Examining the correlation between animal weight and phalanx shape could provide more scientific insights into this hypothesis. Furthermore, exploring asymmetric parameters in relation to weight and investigating the asymmetric effects of weight between the fingers could be considered in future studies.

According to the PC3 results, discrimination between hindlimb and forelimb distal phalanges was more effective than with other PC results. The average of the hindlimb distal phalanges had positive PC3 values. A notable shape variation observed in the negative PC3 values was that the processus extensorius was narrow and thin. However, at positive PC3 values, this formation appeared more developed. The fact that the processus extensorius is more developed in the hindlimb aligns with basic information about the movement system. The extensor muscles of the hindlimb attach to the processus extensorius, facilitating the movement of the foot away from the body and allowing the body to move forward. While the primary role of the hindlimbs in four-legged animals during walking is to push the body forward, the forelimbs are mostly used for braking and directing the walk [32,33]. It can be concluded that the extensor muscles of the hindlimbs work harder than the forelimb muscles during walking and running. This difference in the strength of the extensor muscles between hindlimbs and forelimbs is likely to create a morphological difference in the place where the muscle attaches. In this study, the processus extensorius, to which the extensor muscle attaches, exhibited a thin surface area in the forelimb distal phalanges, whereas in the hindlimb, it inserted onto a larger area.

Distal phalanges play a pivotal role in transferring an animal’s weight to the ground, prompting speculation about the potential influence of weight on their shape. The findings of this study lend credence to the notion that weight exerts an effect on the morphology of distal phalanges. Notably, a correlation between size (CS values) and shape was established, indicating a connection to PC1 and PC3 values. As size increases, the distal phalanx undergoes a lengthening in shape, suggesting a transformative adaptation as weight augments. This alteration in bone shape, concurrent with the escalation of weight, has the potential to distribute the load across a broader area, thereby mitigating the concentration of weight at a single point on the bone. It is crucial to note, however, that while suggestive, the study results offer limited empirical evidence to unequivocally assert this hypothesis. Future investigations could be directed towards substantiating this claim, exploring size and bone density in tandem. Additionally, delving into the biomechanical aspects of how bone density may impact shape could yield more comprehensive and scientifically rigorous results.

In this study, the shape variations of the distal phalanges of Holstein cattle were revealed, and a reference study on this subject was conducted. Although the study results only include reference data for Holstein cattle, these findings could also be applicable to other cattle breeds, revealing differences between breeds. Additionally, geometric morphometry methods and shape analysis studies can be useful in terms of taxonomy, providing valuable information in fields such as zoology and paleozoology.

## 5. Conclusions

In the study, the geometric method was employed to investigate whether distal phalanx groups differed based on their positions. Although they generally exhibited close variations, PC1 statistically differentiated the HL and FM in shape. PC2 distinguished the HL from other distal phalanx groups, while PC3 separated the forelimb and hindlimb groups. The shape (Procrustes distance) of the HM displayed markedly less variability than the other three phalanges. The smallest distal phalanx in size was the HL. For both the forelimb and hindlimb, the medial distal phalanges were larger than the lateral ones. Size (CS) was found to have an effect on PC1 and PC3.

Geometric morphometry was employed to analyze bone shapes concerning gender. Despite the frequent observation of size differences between males and females in numerous studies, consistent shape distinctions have been notably absent. In this study, a deliberate decision was made to overlook gender differences, exclusively opting for male samples of identical age to guarantee research homogeneity. This intentional approach aimed to eliminate both gender and age variations, allowing for a more focused exploration of the specific factors under investigation.

The comprehensive review of finger bones in animals involves referencing linear studies for comparative analysis. Previous investigations have primarily focused on scrutinizing lengths and angular values between specific points, contributing significantly to the expanding body of literature in this domain. Departing from traditional approaches, the current study employs the geometric morphometry method to meticulously unravel the nuanced shape differences exhibited by phalanges. Integrating shape variations alongside conventional linear measurements promises a more nuanced comprehension of the intricate anatomy of distal phalanges. To facilitate this exploration, a meticulously crafted reference model of the same species aims to elucidate the distinct shape variations inherent in diverse distal phalanges within the species. While the present study lays the groundwork for understanding intraspecific morphological differences, prospective research endeavors may be directed towards formulating working hypotheses, potentially involving the comparison of phalanx distalis samples sourced from various breeds. Such future investigations hold the potential to furnish valuable insights, particularly in the realms of veterinary anatomy and taxonomy.

## Figures and Tables

**Table 1:** The mean values for PC1, PC2, and PC3.

PC	HL	HM	FL	FM
PC1	−0.0055	0.0025	0.0139	0.0194
PC2	−0.0016	−0.0168	−0.0246	−0.0241
PC3	0.0422	0.0485	0.0195	0.0144

FL—forelimb lateral distal phalanx; FM—forelimb medial distal phalanx; HL—hindlimb lateral distal phalanx; HM—hindlimb medial distal phalanx.

## Data Availability

The data presented in this study are available on request from the corresponding author (O.G.).
